# Prenatal Exome Sequencing Analysis in Fetuses with Various Ultrasound Findings

**DOI:** 10.3390/jcm13010181

**Published:** 2023-12-28

**Authors:** Antoni Borrell, Elena Ordoñez, Montse Pauta, Juan Otaño, Fernanda Paz-y-Miño, Mafalda de Almeida, Miriam León, Vincenzo Cirigliano

**Affiliations:** 1 Barcelona Centre for Maternal-Fetal and Neonatal Medicine (BCNatal), Hospital Clínic Barcelona, Universitat de Barcelona, 08007 Barcelona, Spain; juan.otano@hospitalitaliano.org.ar (J.O.); mfpaz@clinic.cat (F.P.-y.-M.); 2 Veritas Genetics, c/Zamora 46-48, 08005 Barcelona, Spain; elena.ordonez@veritasint.com (E.O.); mafada.dealmeida@veritasint.com (M.d.A.); miriam.leon@veritasint.com (M.L.); v.cirigliano@gmail.com (V.C.); 3 Institut d’Investigacions Biomèdiques August Pi i Sunyer (IDIBAPS), 08036 Barcelona, Spain

**Keywords:** Exome Sequencing, chromosomal microarray analysis, copy number variations, incidental findings, secondary findings

## Abstract

Objectives: To evaluate the use of Exome Sequencing (ES) for the detection of genome-wide Copy Number Variants (CNVs) and the frequency of SNVs-InDels in selected genes related to developmental disorders in a cohort of consecutive pregnancies undergoing invasive diagnostic procedures for minor or simple ultrasound findings with no indication of ES. Methods: Women undergoing invasive diagnostic testing (chorionic villus sampling or amniocentesis) for QF-PCR and chromosomal microarray analysis (CMA) due to prenatal ultrasound findings without an indication for ES were selected over a five-month period (May–September 2021). ES was performed to compare the efficiency of genome-wide CNV detection against CMA analysis and to detect monogenic disorders. Virtual gene panels were selected to target genes related to ultrasound findings and bioinformatic analysis was performed, prioritizing variants based on the corresponding HPO terms. The broad Fetal Gene panel for developmental disorders developed by the PAGE group was also included in the analysis. Results: A total of 59 out of 61 women consented to participate in this study. There were 36 isolated major fetal anomalies, 11 aneuploidy markers, 6 minor fetal anomalies, 4 multiple anomalies, and 2 other ultrasound signs. Following QF-PCR analysis, two uncultured samples were excluded from this study, and six (10%) common chromosome aneuploidies were detected. In the remaining 51 cases, no pathogenic CNVs were detected at CMA, nor were any pathogenic variants observed in gene panels only targeting the ultrasound indications. Two (3.9%) monogenic diseases, apparently unrelated to the fetal phenotype, were detected: blepharo-cheilo-odontic syndrome (spina bifida) and Duchenne muscular dystrophy (pyelocaliceal dilation). Conclusions: In our series of pregnancies with ultrasound findings, common aneuploidies were the only chromosomal abnormalities present, which were detected in 10% of cases. ES CNV analysis was concordant with CMA results in all cases. No additional findings were provided by only targeting selected genes based on ultrasound findings. Broadening the analysis to a larger number of genes involved in fetal developmental disorders revealed monogenic diseases in 3.9% of cases, which, although apparently not directly related to the indications, were clinically relevant.

## 1. Introduction

Congenital defects include fetal structural defects and genetic disorders. Separately, each group may account for about 3% of the general pregnancy population, although, in several cases, both conditions overlap [1]. Currently, there is an increasing proportion of fetal structural anomalies associated with genetic disorders, which, remarkably, may change the fetal prognosis because little data on neurodevelopment may be extracted from a fetal ultrasound. For many decades, the karyotype was the genetic test most often used for fetal structural defects, affording a 14% diagnostic yield [2]. Over the last decade, chromosomal microarray analysis (CMA) expanded the spectrum of anomalies analyzed to small segmental imbalances beyond the resolution limits of the karyotype, thus becoming the elective choice for prenatal diagnosis in fetuses with structural abnormalities. The incremental diagnostic yield of CMA has been determined to be 8% for all fetal structural anomalies [3]. In more recent years, prenatal Exome Sequencing (ES) has afforded an additional diagnostic yield of 12% above CMA in these pregnancies [4].

CMA has also been established as the elective diagnostic test even in the absence of structural defects [5]; ES is usually performed after CMA, and its use is not recommended in the absence of fetal structural defects, as the fetal phenotype is deemed to be crucial for the interpretation of genomic variants.

This study aims to evaluate the suitability of ES for the detection of Copy Number Variants (CNVs) as a first-line test in prenatal samples, followed by Single Nucleotide Variant (SNV) analysis of virtual gene panels targeted to specific ultrasound abnormalities complemented by a large subset of genes involved in developmental disorders. Pregnancies with isolated or minor fetal structural anomalies undergoing invasive diagnostic procedures for CMA were selected so that the frequency of secondary findings (not directly related to the fetal phenotype) could be evaluated in cases without an established indication for ES.

## 2. Methods

### 2.1. Study Design and Participants

This was a prospective study conducted at the Hospital Clinic Barcelona over a five-month period, where consecutive pregnant women with fetal ultrasound findings undergoing an invasive diagnostic procedure for CMA analysis were invited to participate. Only cases with no indication for ES were selected, including isolated major fetal anomalies, aneuploidy markers, minor fetal anomalies, and other ultrasound signs. Multisystem or recurrent anomalies, skeletal dysplasias, hydrops, hyperechogenic kidneys, complex neurological anomalies, craniosynostosis, and cardiac rhabdomyoma, which are direct indications for ES in our center, were excluded from this study [6].

### 2.2. Sample Collection

Following the invasive diagnostic procedures, small aliquots of amniotic fluids (4 mL) or chorionic villus sampling (CVS) (2–3 fronds) were separated from the whole sample before being sent to the Biomedical Diagnostic Center laboratory of the Hospital Clinic Barcelona, where clinical diagnostic tests (Quantitative Fluorescent Polymerase Chain Reaction (QF-PCR) and CMA) were performed. Anonymized aliquots were sent to the Veritas Genetics laboratory (Barcelona, Catalonia, Spain) for ES, with a detailed description of the fetal phenotype and/or ultrasound findings, including the human phenotype ontology (HPO) terms to be used in the bioinformatic analysis pipeline for variant prioritization [7].

### 2.3. QF-PCR Analysis

Genomic DNA was isolated from chorionic villi or amniotic fluids using the Qiagen Mini Kit (Qiagen, Valencia, CA, USA) following the manufacturer’s protocol. Before CMA or ES, extracted DNA was tested in both laboratories through QF-PCR assays for rapid prenatal diagnosis of aneuploidies of chromosomes 13, 18, 21, X, and Y and to exclude the presence of maternal cell contamination, as previously described [8].

### 2.4. Chromosomal Microarray Analysis

CMA was performed using an oligonucleotide array targeted to regions associated with clinically recognized syndromes (qChipCM, 8 × 60 K, qGenomics, Esplugues de Llobregat, Barcelona, Spain)). This array has a backbone resolution of 350–500 Kb, increasing to ~100–125 Kb in subtelomeric and pericentromeric regions and ~30 Kb in regions associated with constitutional pathology. Captured images were quantified and analyzed using qGenviewer software v2.1.1(qGenomics).

### 2.5. Exome Sequencing

Extracted DNA was processed through enzymatic digestion to build paired-end sequencing libraries. After pooling, these were captured to enrich for the coding regions of 19,443 genes using a highly customized probe set (Nonacus, Quinton, UK). The probe design included coverage enhancement for all OMIM morbid genes and regions associated with developmental anomalies, additional non-coding disease-causing variants, and extended probe distribution to allow genome-wide and exon-level detection of CNVs. 

Enriched library pools were sequenced on NovaSeq 6000 instruments (Illumina, San Diego, CA, USA) at a mean depth of 100×, sequencing outputs were analyzed with dedicated software that allows CNV calling, and SNV filtering was performed according to allele frequency, effect on protein, and prioritization based on HPO terms (Congenica, Hinxton, UK). All samples were tested blind, CNV analysis was performed on sequencing data as a first analysis step, and the results were compared with the corresponding CMA. CNVs below 200 Kb were filtered out unless present in regions of known dosage sensitivity or in genes included in virtual panels, which were analyzed at single-exome resolution.

Virtual multigene panels were used to filter for selected genes to target specific ultrasound indications; additionally, the broad virtual gene panel from the PAGE (Prenatal Assessment of Genomes and Exomes) group [9], targeting 1542 genes related to developmental disorders, was also applied in all cases. An additional panel designed for comprehensive neonatal screening, including 407 genes related to early onset, actionable diseases, was performed if fetuses were born during the turn-around time of CMA. Variants were classified according to the American College of Medical Genetics (ACMG) criteria [10], and only pathogenic or likely pathogenic variants were reported. Filtering and prioritization were performed according to allele frequency and the effect on protein.

### 2.6. Ethical Approval

This study was approved by the Internal Review Board (IRB) of the Hospital Clinic Barcelona (IRB number HCB/2021/0271). All pregnant women participating in this study gave their written informed consent, in which they could opt to be informed of eventual incidental findings. Pregnancy outcomes were obtained by reviewing the medical records.

## 3. Results

A total of 59 consecutive pregnant women undergoing an invasive procedure for fetal ultrasound findings with no indication for ES consented to participate in this study and to be informed of incidental findings. Isolated major fetal anomalies were present in 36 cases, 11 aneuploidy markers, 6 minor fetal anomalies, 4 multiple anomalies, and 2 other ultrasound signs. Common aneuploidies were detected through QF-PCR in six (6/59; 10%) pregnancies (two trisomies 21, two trisomies 13, one trisomy 18, and one double trisomy X and 18), all in fetuses with aneuploidy markers and one with multiple anomalies (Table 1), and were excluded from further analyses. Two other uncultured samples had high levels of maternal cell contamination and were also excluded from this study. 

The remaining 51 cases were studied at a mean gestational age of 21 + 3 weeks (range 11–33 weeks); all produced normal CMA results and underwent ES, which was successful in all cases and did not detect any additional pathogenic CNV (100% concordance). 

No relevant findings were observed through the ES analysis of multigene panels selected to target ultrasound indications. Pathogenic variants in additional genes included in the PAGE panel were found in two (3.9%) cases: blepharo-cheilo-odontic syndrome (in a fetus with spina bifida) and Duchenne muscular dystrophy (in a fetus with bilateral pyelocaliceal dilation)(Table 2). The two causative diagnoses were achieved in fetuses with non-complex major anomalies (Table 1), accounting for 5.6% of cases with non-complex anomalies. In one case of hyperechogenic bowel, ES was completed after birth, and the analysis further extended to genes related to early onset actionable disorders, revealing a neonatal G6PDH deficiency.

Case #1: A 33-year-old pregnant woman in her first pregnancy with a previous history of septoplasty due to bicorn uterus and first-trimester screening for aneuploidies at low risk. At the 20-week scan, lumbosacral myeloschisis ranging from L5 to low sacrum was detected, associated with bilateral 10 mm ventriculomegaly and lemon and banana signs. Parents opted for pregnancy termination, and amniotic fluid was collected before the procedure at 21 w showing normal QF-PCR and CMA results.

Post-mortem evaluation confirmed myeloschisis L4-S5 and brain changes compatible with the Arnold–Chiari spectrum. ES detected a splicing variant (within ± 2 bp of the splice site) in the *CTNND1* gene (NM_001085458):c.2092-2A>T classified as pathogenic. Loss of function of *CTNND1* is a known mechanism of disease associated with blepharo-cheilo-odontic syndrome 2 of autosomal dominant inheritance, incomplete penetrance, and phenotypic variability. The mother was found to be a carrier of this variant without any apparent signs associated with this syndrome. Interestingly, while neural tube defects have been associated with blepharo-cheilo-odontic syndrome 1 caused by variants in the *CDH1* gene, to date, an association with *CTNND1* has not been reported.

Case #2: A 30-year-old woman with a previous obstetric history of an early pregnancy loss, with normal ultrasound, and low risk at first-trimester screening for aneuploidies. Moderate bilateral pyelocaliceal dilation was observed at 36 weeks (8.7 mm in the right and 9.6 mm in the left kidney) associated with mild polyhydramnios. Amniocentesis was performed with normal QF-PCR and CMA results. After labor induction at 41 weeks, a male infant was delivered weighing 3240 g and presenting 9–10 Apgar scores.

ES analysis resulted in hemizygosity for a truncation variant of the *DMD* gene (NM_004006.2):c.3922-2A>G, classified as likely pathogenic in relation to X-linked dystrophinopathies. This variant affecting splicing was absent in the general population database (GnomAD), nor was it described in ClinVar. In silico predictors classified it as deleterious and associated with the Duchenne phenotype in Varsome. Parental segregation analysis confirmed the mother to be a carrier of this variant. The ES result was delivered to the family postnatally, and the newborn was referred to a muscular dystrophies clinic, where the variant was confirmed and creatine phosphokinase (CPK) values were not excessively high.

## 4. Discussion

We studied a series of 51 consecutive pregnant women undergoing an invasive procedure due to fetal structural defects or ultrasound findings that fulfilled criteria for CMA. ES analysis of virtual multigene panels only targeting ultrasound markers was not found to be useful for increasing diagnostic yield in these cases. Broadening the analysis to the wider PAGE multigene panel [9], although unrelated to the ultrasound findings, resulted in a 3.9% (2/51) rate of monogenic disorders, which increased to 5.6% (2/36) of cases if only the group of non-complex major structural anomalies is considered. The two disorders were blepharo-cheilo-odontic syndrome (spina bifida) and Duchenne muscular dystrophy (hydronephrosis). This is a high rate of variants in known disease genes related to developmental disorders, which, although not directly associated with referrals, can be deliberately analyzed through ES and detected as secondary findings.

Interestingly, fetal aneuploidies were found through QF-PCR in fetuses with ultrasound markers and monogenic disorders in those with non-complex major anomalies. No CNVs were found in this series with the two different methods used, standard CMA and ES, which afforded fully concordant results, one of the initial main aims of this study. Recent advances in the early detection of fetal anomalies already in the first trimester, including spina bifida, could also improve the management of invasive testing and allow more time for multiple analyses (karyotype, CMA, ES) and counselling for the parents, especially in countries with a limited period for pregnancy termination [11].

About one third of prenatal ES studies have reported information about incidental or secondary findings. In 2021, Mellis et al. published a systematic review of 72 studies on prenatal ES, 23 of which reported incidental findings with a 6.3% (129/2062) mean yield, with 5 studies yielding above 10% [12]. One of these groups, Zhu et al., reported 4 (4.4%) incidental findings among 90 pregnancies undergoing trio-based ES incorporating splice-site and mitochondrial genome analysis among fetuses with structural abnormalities [13] All findings had clinical relevance because the four couples chose to terminate their pregnancies [14].

Whether incidental findings should be reported is controversial [1]. The ISPD guidelines state that “there is no universal consensus on the management of incidental findings and each center should convey their policy detailing whether they are or are not reported, and if reported what is included for parents and fetus” [15]. The ACMG guidelines state that “highly penetrant pathogenic variants detected in genes unrelated to the fetal phenotype, but known to cause moderate to severe childhood-onset disorders, are recommended to be reported” because “many of these disorders, especially those associated with non-syndromic intellectual disability/neurodevelopmental disorders and metabolic conditions, are not detectable with fetal imaging” [16]. The Belgian guidelines state that late-onset disorders with clinical utility, such as variants causing late-onset disorders, typically cancer caused by mutations of a tumor-suppressor gene, will be communicated if an undeniable health benefit can be expected in addition to carriership for X-linked recessive disorders, while late-onset disease that is not actionable and carriership for autosomal recessive disorders will not be reported [17].

In contrast, the British National Health Service guidelines state that “testing will focus on identifying disease-causing variants of direct relevance to the clinical referral and additional findings will not be actively sought. The testing strategy aims to reduce the likelihood of identifying pathogenic variants that predispose to other rare diseases but the possibility of incidental findings cannot be excluded. Such findings may be discussed with the referring clinician on a case-by-case basis” [18].

When ES is applied in low-risk pregnancies upon parental request, all pathogenic and likely pathogenic variants are secondary or incidental. While this practice is not recommended by any guideline, Vaknin et al. applied an ES-based multigene panel including early-onset severe genetic disorders in 210 pregnancies with no fetal structural defects divided into two subgroups: 50 cases with minor ultrasound findings (e.g., mildly echogenic bowel, absent ductus venosus, femoral length between the 5th and 25th percentile, and head circumference between the 5th and 15th percentile) and 160 cases without any suspicious ultrasound findings. They found pathogenic or likely pathogenic variants in 2.9% (6/210) of cases overall, divided into 10% (5/50) for minor ultrasound findings and 0.6% for absent findings [19]. They concluded that their results showed a high rate of abnormal findings with ES, even in apparently normal pregnancies. These results are discordant with ours because we found a monogenic disorder in two fetuses with major ultrasound findings, spina bifida and bilateral pyelocaliceal dilation, accounting for a 5.7% (2/36) yield in major structural anomalies, while fetal aneuploidies were revealed in fetuses with ultrasound aneuploidy markers.

There are some limitations to our study. First, the sample size is limited, as only 61 pregnancies were included. Second, there are some limitations inherent to the short reads sequencing approach, such as the inability to detect variants in deep intronic regions and a limited capacity to accurately analyze repeat sequences and areas with a high GC content. Another limitation of sequencing studies is that while pathogenic variants, as in case #1, carry a 99.9% certainty of being associated with the disease, likely pathogenic variants, as in case #2, carry a higher than 90% certainty. On the other hand, sequencing allowed for performing CNVs as a first analysis step of ES, thus lowering the time for both analyses to two weeks compared with the sequential use of CMA before ES.

To conclude, the performance of ES with wide filtering for developmental disorders in fetuses with no direct indication for ES appears to be useful, given that, although unrelated to the fetal phenotype, monogenic disorders can be detected in 3.9% or up to 5.6% of cases.

## Figures and Tables

**Table 1:** Genetic anomalies found with the different genetic tests applied in each of the five groups studied, defined according to the type of ultrasound findings.

Ultrasound Findings Group	N	Aneuploidies	Maternal Cell Contamination	Included Cases	CNV	SNV
Major structural defects	36	0	1	35	0	2 (5.6%)
Minor structural defects	6	0	0	6	0	0
Aneuploidy markers	11	5 (45%)	1	5	0	0
Multiple findings	4	1	0	3	0	0
Other	2	0	0	2	0	0
Total	59	6 (10%)	2	51	0	2 (3.9%)

**Table 2:** Variants found through Exome Sequencing and their classification according to various databases.

	Fetal Phenotype	Gestational Age	Pregnancy Outcome	Variant	Gene	Variant Type	GnomAD	ClinVar	Varsome	Franklin
CASE#1	Lumbosacral myeloschysis (L5 to low sacrum)	21 + 3	Termination of pregnancy	NM_001085458: c.2092-2A>T	*CTNND1*	Splicing variant	Variant not found	No data	L. Pat	L. Pat
CASE#2	Moderate bilateral pyelocaliceal dilation (8.7 mm and 9.6 mm), mild polyhydramnios	36 + 2	Alive and well	NM_004006.2: c.3922-2A>G	*DMD*	Splicing variant	Variant not found	No data	L. Pat	L. Pat

## Data Availability

The data presented in this study are available on request from the corresponding author. The data are not publicly available due to privacy restrictions.
